# Outcomes 10-years after traumatic spinal cord injury in Botswana - a long-term follow-up study

**DOI:** 10.1038/s41394-024-00671-0

**Published:** 2024-08-07

**Authors:** Inka Löfvenmark, Wame Mogome, Kobamelo Sekakela

**Affiliations:** 1 Karolinska Institutet, Institution for neurobiology, care science and society, Stockholm, Sweden; 2 Spinalis Foundation, Solna, Sweden; 3 Spinalis Botswana SCI-rehabilitation Centre, Gaborone, Botswana

**Keywords:** Outcomes research, Health services

## Abstract

**Study design:**

Prospective follow-up study.

**Objectives:**

To describe outcomes, survival, and attendance to routine follow-up visits 10 years post-SCI.

**Setting:**

The national SCI-rehabilitation center in Botswana.

**Methods:**

All persons who were admitted with traumatic SCI during a 2-year period, 2011–2013, and survived up to 2 years post-injury were included. Data were collected from the medical records from the follow-up assessment closest to 10 years post-SCI and included demographic and clinical characteristics, functional outcomes, and secondary complications. Data regarding mortalities were received from relatives. Statistical comparisons were made, when possible, between those who attend follow-up assessment and those who did not, and between those who survived up to 10 years post-SCI and those who died.

**Results:**

The follow-up rate was 76% (19/25) of known survivors. No statistically significant factors were found to affect the follow-up rate. Secondary complications rates were for pressure ulcers and urinary tract infections 21%. Self-catheterisation and suprapubic catheter were the preferred methods to manage neurogenic bladder dysfunction. Ten persons (26%) had deceased since 2^nd^ follow-up assessment. The causes of death were probably SCI-related in more than half of the cases.

**Conclusions:**

This was a follow-up study at year 10 after acute TSCI in Botswana conducted at the national SCI-rehabilitation center. The study supports previous reports regarding the importance of that having specialized SCI units and the need of structured follow-ups, a responsible person in charge of scheduling, and updated patient registers. We found high follow-up rate, low rates of complications and of patients being lost to follow-up.

## Background

Living with spinal cord injury (SCI) imposes an increased vulnerability for preventable complication leading to increased suffering, and increased risk of morbidity and premature death, especially in low-resource settings [1, 2]. The life expectancy for persons with SCI have improved greatly during the last decades in many industrialized countries [1, 3–6], attributed to the development of specialized SCI-care and structured long-term follow-ups. To be managed at a specialized unit after SCI is beneficial both for the patient and for the society’s economy [7].

Structured follow-ups are conducted as comprehensive interdisciplinary re-evaluation with the main aim to prevent complications and decrease morbidity and mortality [8]. The first year’s follow-up assessments often focus on facilitating reintegration in communities and return-to-work, while in the long run the focus changes to health maintenance, preventing the needs for re-admissions, and decreasing health care cost [8]. Specialized rehabilitation services and follow-up after discharge is often non-existing in low-resourced settings [1, 5, 9]. Even when rehabilitation centers are available, routines for follow-ups are often lacking and mortality rates are substantially higher compared with more resourceful settings [1]. Patients are often lost to follow-up and their status is unknown [10, 11]. Lack of patient registers and contact details, vast distances to specialized health care clinics, and lack of transport are some reasons for not adequate follow-ups [9].

In many low-resource settings, preventable secondary complications such as sepsis due to pressure ulcers (PU) [5, 12, 13] or recurrent urinary tract infections (UTI), and kidney and respiratory failure [9, 10] lead to high mortality rates in the SCI-population [3]. Several studies have also shown an increased risk for cardiovascular disease in the SCI-population [1, 8, 9, 14] which constitute a common cause for mortality in high-income countries [6].

Botswana is a middle-income country in southern Africa. Persons who sustained a traumatic SCI (TSCI) are transferred to the national referral hospital, Princess Marina Hospital (PMH) in the capital, and the SCI-rehabilitation center. The center was established in 2010 through a 3-year partnership project between the Ministry of Health in Botswana and the Spinalis Foundation in Sweden, which was partly funded by the Swedish International Development Cooperation Agency (Sida). Since 2013 the unit has been run by local professionals only and has provided in-patient services, re-rehabilitation, out-patient clinics, and structured long-term follow-up. The follow-ups, conducted as interdisciplinary assessments, are an important part of the structure of the center and are to be conducted at a minimum at 1^rst^, 2^nd^, 5^th^ year post-injury and thereafter every fifth year. Often an early assessment is included due to the known high risk of developing complications and premature death within one year after discharge [1, 3, 8]. Urine and blood samples, such as kidney and liver function, are routinely conducted during the follow-up visit. Patients are scheduled by the responsible nurse and those living far away are offered lodging at the center. The up-take area of the national center is the whole country, i.e., some persons have to travel approximately 900 km one way.

### Summary of the previous studies on this patient group

Three studies were previously conducted on the same cohort as in the current study, namely all persons admitted with acute TSCI during a 2-years period. These studies followed three phases within the chain of SCI-care: acute admission, discharge, and follow-up two years post-injury. The main results of the studies are presented below, for detailed information, articles are published in Spinal Cord.

Acute admission [15]: 52 persons with acute SCI were admitted; however, three persons did not consent therefore 49 participants were included, 71% male, age range was 4–81 years with 80% ⩽45 years, 59% had tetraplegia with 39% having a high tetraplegia (C1–C4 level), mainly incomplete. Causes of injury were traffic related (68%), assault (16%), and falls (10%). Mortality prior to admission to the SCI-rehabilitation center was 20%, where all, but one, had tetraplegia, resulting in that 39 persons were admitted for rehabilitation.

At discharge [16]: 38 persons were discharged after completed rehabilitation; one person deceased prior to discharge after having completed rehabilitation. Median length of stay was 20 weeks with complete injuries and presence of PU being the factors that mostly prolonged hospitalization. Clean intermittent catheterization (CIC) or suprapubic catheter (SPC) were the main methods for bladder management and digital ano-rectal stimulation for bowel management. The most frequent complications during admission were PU, UTI, and pain.

*At 2*^*nd*^ follow-up [17]: follow-up rate was 71% (27 out of 38 persons attended follow-up, the remaining were contacted by phone), with higher attendance among those with complete injuries and those with secondary complications. Age, gender, distance to the center, or education did not affect the follow-up rate. CIC and SPC remained the preferred methods of bladder management. Despite high rates of PU and UTI, no deaths had occurred during the follow-up period resulting in 38 persons survived to be included in the current study.

### Rational

Botswana, as well as a substantial part of Sub-Saharan Africa, is lacking reliable information on the long-term outcome and mortality after SCI [9, 10, 18]. After initial establishment of a specialized SCI-center in Botswana, outcomes for persons with TSCI improved and mortality was decreased for up to two years post-injury [17]. Complications were still prevalent, however, at a lower rate compared with before the SCI-center. Therefore, the aim of this study is to describe the long-term outcomes 10 years post-TSCI in Botswana after completing rehabilitation and attending to the follow-up assessments.

Study goals:

1. To evaluate the procedures of interdisciplinary follow-up assessments at the SCI-center.
2. To describe the medical status, consequences, and complications 10 years post-SCI for persons with TSCI in Botswana.
3. To describe home situation, functional outcomes, quality of life, and prevalence of work/studies 10 years post-SCI.
4. To describe rate and causes of mortality for persons with TSCI after the 2^nd^ yearly follow-up and whether causes of death were SCI-related.

## Materials and methods

**Fig. 1:**
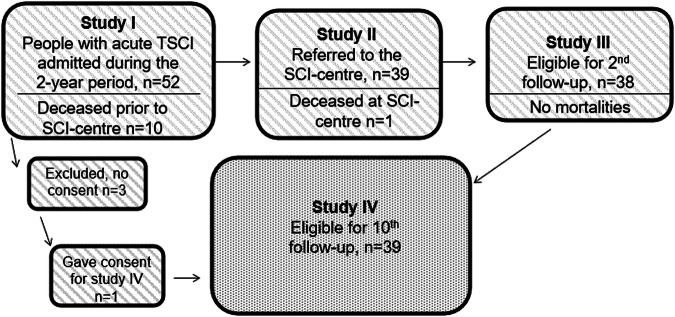
Flowsheet on included and deceased participants in the four consecutive studies on the same population of patients admitted at Princess Marina Hospital in Botswana. Study I-III are previously published, study IV is the current study.

This was a prospective follow-up study conducted at the national SCI-rehabilitation center in Botswana. The study population includes all persons admitted to PMH with acute TSCI between February 1^rst^ 2011 – January 31^rst^ 2013 and who had survived up to two years post-SCI, i.e. the same cohort as in the three previous studies, with an addition of one person who did not consent initially, but now gave verbal consent to participate, resulting in 39 participants who were included in the current study (Fig. 1).

### Data collection

Data were collected from the medical files from the follow-up assessment as close as possible to the follow-up at 10 years post-SCI. Protocols used at the assessments are mainly derived from the International Spinal Cord Society (ISCoS) [19] partly adapted to this setting. The following protocols were used:

1. International standards for neurological classification of SCI [20]
2. ISCoS data sets:
  - CardiovascularMedical history regarding cardiac and circulatory problems. Questions added to the data set; pulmonary diseases, tumors, tropical diseases, diabetes, HIV/AIDS.
  - Pain
  - Lower urinary tract function
  - Bowel function
  - Sexual function
  - Quality of life (QoL): Includes three questions; ‘how satisfied are you’ with the following: ‘life as a whole’, ‘physical health’, and ‘psychological health, emotions and mood’. Ratings 0–10 completely dissatisfied–completely satisfied.
3. Sociodemographic protocol: Sociodemographic, work/study
4. Functional Independence Measure (FIM)
5. Pressure ulcer data: Previous and current pressure wounds.

Data regarding mortality rate, time, place, and causes of deaths have mainly been retrieved from the relatives since most persons deceased in their homes and no data regarding mortality were available. An autopsy is only conducted at the request of the family when a person dies in their home. Data for deceased participants have been retrieved from their latest follow-up assessment.

### Analysis

IBM SPSS statistics 28.0.0.0 (Armonk, NY, USA) were used for analyzing data. Categorical variables are presented as absolute numbers and proportions, and continuous variables as mean and standard deviation (SD), and median and interquartile range (IQR) [21]. Non-parametric tests were used for comparing groups because of the small sample, Mann–Whitney U-test for continuous variables and Fisher’s exact test for categorical variables. *P*-value were set to *P* < 0.05.

Time frame: the follow-ups at year 10 were to be conducted between February 2021 and March 2023. Data collection from patient files, analysis, writing manuscript, and publishing was conducted in August 2023 – January 2024.

## Result

Out of the 39 participants who were included in this study, at least 25 survived up to 10 years, 10 participants had deceased, and four were lost to follow-up and their status is unknown. Demographic and clinical characteristics are presented in Table 1.

**Table 1:** Demographic and clinical characteristics and functional outcomes of persons who survived until follow-up visit at year 10 post-SCI, attended/not attended the follow-up, persons who deceased after two years post-injury, and persons lost to follow-up.

Variables	Survived to follow-up at year 10, (*n* = 25)			Deceased prior to year 10 follow-up (*n* = 10)	*P*-value between survivors and deceased	Lost to follow-up (*n* = 4)
	Attended year 10 follow-up (*n* = 19)	Not attended year 10 follow-up (*n* = 6)	*P*-value between attending year 10 follow-up or not attending			
Demographics						
*Age at injury (years)*			0.780^a^		0.090^a^	
Mean (SD)	29.6 (11.12)	28.5 (9.93)		37 *(11.32)*		32.5 (18.67)
Median (IQR)	26 (22–38)	29 (18–35)		32.5 (30–48)		30 (13.5–54)
*Gender* *n* (%)			0.624^b^		0.235^b^	
Male	12 (63)	5 (83)		9 (90)		1 (25)
Female	7 (37)	1 (17)		1 (10)		3 (75)
Socio-demography						
*Marital status* *n* (%)						
Single	11 (58)	1 (17)		4 (40)		–
Married-cohabit	5 (26)	–		2 (20)		–
Divorced	2 (11)	–		–		–
Child	1 (5)	0		0		0
Missing data	0	5 (83)		4 (40)		4 (100)
*Live/d with* *n* (%)						
Family/parents/siblings	8 (42)	1 (17)		2 (20)		–
Spouse	4 (21)	–		2 (20)		–
Relative/s	1 (5)	–		2 (20)		–
Alone	2 (11)	–		–		–
Kids	3 (16)	–		–		–
Institution home/work	1 (5)	–		–		–
Missing data	0	5 (83)		4 (40)		4 (100)
Clinical characteristics						
*Severity of injury* *n* (%)			0.538^b^		0.561^b^	
Tetraplegia AIS A,B,C	4 (21)	2 (33)		3 (30)		0
Paraplegia AIS A,B,C	10 (53)	1 (17)		6 (60)		0
AIS D	5 (26)	2 (33)		1 (10)		4 (100)
Missing data	0	1 (17)		0		0
*Level of lesion* *n* (%)			1.000^b^		1.000^b^	
Tetraplegia	8 (42)	3 (50)		4 (40)		3 (7 5)
Paraplegia	11 (58)	3 (50)		6 (60)		1 (25)
*Cause of SCI* *n* (%)			0.480^b^		0.063^b^	
Traffic-related	13 (68)	3 (50)		6 (60)		1 (25)
Assault	3 (16)	3 (50)		0		2 (50)
Falls	1 (5)	0		3 (30)		1 (25)
Other	2 (11)	0		1 (10)		0
Functional outcomes						
*Mode of mobility* *n* (%)			0.091^b^		0.399^b^	
Wheelchair						
Active manual	10 (53)	0		5 (50)		0
Electric	1 (5)	1 (17)		0		0
Both manual and electric	1 (5)	1 (17)		3 (30)		0
Ambulatory + wheelchair	3 (16)	1 (17)		1 (10)		0
Ambulatory	4 (21)	3 (49)		1 (10)		4 (100)
*Work* *n* (%)						
Yes	7 (37)	1 (17)		2 (20)		–
No	10 (53)	–		4 (40)		–
Student	2 (10)	–		–		–
Missing data	0	5 (83)		4 (40)		4 (100)
*Bladder management* *n* (%)						
Self-catheterization	4 (21)	–		3 (30)		–
Suprapubic catheter	5 (26)	1 (17)		2 (20)		–
Indwelling catheter	3 (16)	–		–		–
Normal	7 (37)	–		–		–
Condom catheter	0	–		1 (10)		–
Missing data	0	5 (83)		4 (40)		4 (100)
*Bowel management* *n* (%)						
Digital stimulation	9 (47)	1 (17)		3 (30)		–
Colostomy	3 (16)	–		1 (10)		–
Normal	7 (37)	–		1 (10)		–
Missing data	0	5 (83)		5 (50)		4 (100)
FIM scores						
Mean (SD)	79.21 (12.33)	–		67 (24.495)	0.121^a^	–
Median (IQR)	84 (70–86)	–		78.50 (49–82.50)		–
Missing data	0	5		4		4
FIM scores divided by injury (Mean (SD))						
Tetraplegia AIS A,B,C	67.75 (20.11)					
Paraplegia AIS A,B,C	79.5 (8.0)					
All AIS D	(87.8 (3.56)					
*Quality of Life*						
Life as a whole Mean (SD)	7.63 (2.36)	–		8.17 (2.86)	0.427^a^	–
Median (IQR)	8 (6–10)			10 (5–19)		
Physical health Mean (SD)	8.05 (2.74)	–		6.33 (4.32)	0.555^a^	–
Median (IQR)	10 (5–10)			7.5 (3–10)		
Psychological health Mean (SD)	8.32 (2.24)	–		6.83 (4.92)	1.000^a^	–
Median (IQR)	9 (5–10)			10 (1–10)		
Missing data	0	6		4		4

*AIS* American spinal injury association Impairment Scale, *FIM* Functional Independence Measure, *IQR* interquartile range, *SCI* spinal cord injury, *SD* standard deviation.

^a^Mann–Whitney U-test.

^b^Fisher’s exact test (2-sided).

Of the 25 persons eligible for structured follow-up visits, 19 were scheduled and assessed which equal to a 76% follow-up rate. The time from date of injury to the follow-up visits closest in time to 10 years post-SCI varied between 68–141 months, with a mean (SD) of 113.8 (22.4).

Reasons for not attending follow-up included: not wanting or feeling a need for follow-up (*n* = 2), could not be scheduled due to incorrect contact information (*n* = 2), living far away and having to take time off from work (*n* = 1) (living 900 km away from the clinic), and one participant did not have a relative who could drive him to the hospital. No demographic or clinical characteristic factors differed between those who attended or did not attend follow-up. Cause of injury did not show any differences between groups but was included in the analysis due to persons with traffic related injuries are being covered by the Motor vehicle accident-fund which facilitates provision of consumables and technical aids.

The primary reason for persons being lost to follow-up is the lack of contact information. Staff members have repeatedly made efforts to schedule all persons with TSCI, however their contacts have not been going through, and they have not contacted the unit themselves.

For participants who attended follow-up visit (*n* = 19), 10 participants had an AIS A TSCI (Table 1). Five participants had improved AIS-score with 1–3 steps compared with initial assessment.

The prevalence of PU and UTI were 21%. Ten participants reported pain with musculoskeletal pain being most prevalent (Table 2).

**Table 2:** Complications and medical issues at 10 years post-spinal cord injury.

Variables: *n* (%)	Survived to follow-up at year 10 (*n* = 25)		Died prior to 10^th^ follow-up (*n* = 10)	Lost to follow-up (*n* = 4)
	Attended 10^th^ follow-up (*n* = 19)	Not attended 10^th^ follow-up (*n* = 6)		
*Pressure ulcer/s*				
Current or last year	4 (21)^a^	–	3 (30)^b^	–
Missing data	1 (5)	6 (100)	4 (40)	4 (100)
*Urinary tract infections*	4 (21)	–	1 (10)	–
Missing data	0	6 (100)	4 (40)	4 (100)
*Pain*	10 (53)	1 (17)	4 (40)	–
Neuropathic	1 (10)	–	2 (50)	–
Musculoskeletal	5 (50)	1	2 (50)	–
Neuropathic and musculoskeletal	4 (40)	–	0	–
Missing data	0	5 (83)	4 (40)	4 (100)
*Cardiovascular issues*				
Hypertension	6 (32)	–	0	–
Orthostatic hypotension	0	–	1 (10)	–
*Heterotopic ossification* /myositis	1 (5)	–	–	–
*Other non-SCI-related diagnosis*				
HIV	4 (21)	–	2 (20)	–
Diabetes	1 (5)	–	–	–
Tuberculosis	0	–	1 (10)	–
Severe scoliosis	1 (5)	–	–	–
Missing data	0	6 (100)	4 (40)	4 (100)

Data from deceased persons are collected from last performed yearly follow-up assessment.

^a^One had pressure ulcers during in-patient care and one at both in-patient and at 2^nd^ yearly follow-up.

^b^Two had pressure ulcer during in-patient care.

Eleven participants were single, however only two lived alone. The majority lived together with family members such as parents or siblings. Five participants reported their house to be accessible to their needs, however several lived in houses that were not adequately adapted. Ten participants were using manual wheelchairs and seven were partly or only ambulating. FIM and QoL were rated by all participants attending follow-ups with total mean scores of 79 out of maximum 91 and 7.6–8.3/10 respectively (Table 1). We did not find significant differences for QoL compared with the 2^nd^ year follow-up.

Regarding bladder and bowel management, seven participants reported normal function in both areas. Most of the participants used the same methods for management of neurologic bladder as they did at the 2^nd^ follow-up i.e. mainly CIC or SPC (Table 1). One child had progressed from CIC by assistant to self-catheterization.

Digital ano-rectal stimulation was the primary method to manage bowel dysfunction, however seven participants had gone back to performing it on their beds, whereas the rest used the toilet or a commode chair. Three participants had colostomy, of whom one had got it due to non-SCI related issues (Table 1).

All but one child rated sexual function which is presented in Table 3.

**Table 3:** Sexual function rating according to ISCoS data set for sexual function.

Men (*n* = 12)/Women (*n* = 6)^a^	Normal	Reduced/altered	Absent	Unknown
Psychogenic erection/arousal	4/2	4/2	3/1	1/1
Reflex erection/arousal	6/2	1/2	4/1	1/1
Ejaculation (men only)	5	0	6	1
Orgasmic functions	6/1	0/1	5/2	1/2

^a^One child was not applicable to answer, therefore data is presented for 18 persons.

Seven participants were working, 10 were not working (one recently stopped due to medical reasons), and two were students (Table 1).

Among the participants who deceased since the 2^nd^ follow-up visit (*n* = 10) seven had an AIS A injury, four had tetraplegia, and the mean survival time from injury was 5.4 years. Causes of deaths were believed to be SCI-related for at least seven participants with possible causes being sepsis due to PU, collapsed (possibly due to cardiovascular disease), and autonomic dysreflexia. At least two were none-related to SCI, and for one participant cause is unknown. Further data are presented in Table 4 and derives from their last follow-up assessments, conducted 3–6 years after TSCI. Data were largely missing for four persons due to missing patient files.

**Table 4:** Characteristics of persons deceased between yearly follow-up visits at year 2 and 10 (*n* = 10).

Variables	Last follow-up
*Age at death*	
Mean (SD)	42.50 (11.404)
Median (IQR)	39.50 (33.50–55.25)
*Time from SCI to:*	
- Last follow-up visit (months)	
Mean (SD)	48.83 (18.872)
Median (IQR)	38.50 (35.50–72.50)
- Death (years)	
Mean (SD)	5.40 (2.591)
Median (IQR)	5.5 (3.75–7.25)
*Cause of death (n)*	
Pressure ulcer (and HIV)	4 (2)
Autonomic dysreflexia	1
Collapse	2
HIV	1
Road traffic accident	1
Unknown	1
*Place of death (n)*	
Home	8
Hospital/SCI-center	1
Unknown	1

Data collected from the last conducted follow-up assessment.

*IQR* interquartile range, *SCI* spinal cord injury, *SD* standard deviation.

## Discussion

The present study was a follow-up study at year 10 post-SCI in Botswana, including the same participants who were included in three previously published studies [15–17].

Structured long-term follow-up is often a challenge in low-resource settings with high rates of patients being lost to follow-up [5, 11, 22]. Lack of transport is a common problem all over the African region and distances are vast. Therefore, the follow-up rate of 76% and only four persons being lost to follow-up are positive findings. Factors facilitating the follow-up rate is likely to include the existence of the SCI-unit, the structure of interdisciplinary follow-ups, an understanding among the staff that follow-up assessments are valuable, up-dated patient register, and an allocated nurse being responsible for scheduling patients. However, follow-up frequency was inconsistent, with occasional gaps of time between scheduled visits. There are several reasons for these gaps of time of which one is a lack of managing schedule slots due to a rotation system of staff in the public health care system. These rotations of staff have resulted in only one responsible nurse that is still there out of the initial 10 nurses that were trained by the Swedish staff. Continuous training of new staff has been challenged by the large number of rotations and lack of time. When the responsible nurse is not at work, scheduling patients, especially with short notice in the event of cancellation, has not been prioritized and is not efficient. Additionally, the physiotherapy and occupational therapy staff has been severely reduced which is why, at times, the center is without therapists. Other barriers are lack of transport which has also been reported from other settings [9, 11]. Transport can be provided by the local clinics when cars are available, however, it is often an unreliable system due to uncertainty if the car will be available for the transport home again. Previously, out-reach programs, also recommended by Burns et al. [5], were in place with the staff being able to assess persons with SCI in rural settings, but that alternative has become very limited and is not used today for the SCI-team. Finally, during the Covid pandemic, almost no follow-up assessments were conducted as the hospital was one of Botswana’s primary Covid hospitals.

For the 19 participants assessed, the medical status was in general satisfactory with relatively low rates of secondary complications. The rate of complications has decreased compared with the 2^nd^ follow-up [17] where 48% PU and 41% UTI were found. Madasa et al. [22] recently reported 29% PU four years post-injury, however 40% were lost to follow-up making the numbers uncertain. Furthermore, Hossein et al. [23] reported 26% PU after approximately three years post-injury, however over 75% of the wheelchair users reported problems with PU prior to the follow-up. They also address the challenges to prevent PU in the home environment, despite having patient education in their program and provision of wheelchairs and cushions. We attribute the relatively good health among the participant who attended the follow-ups in Botswana to the rehabilitation offered at the unit, including training and provision of appropriate technical aids but also patient and family education. This is in line with Illis et al. [7], who states that having access to specialized SCI-units decreases complications such as PU and UTI. The low rates of complications might also be due to that persons with SCI with time learn prevention strategies at a more robust level and how to care for their bodies in a more optimal way. Just as important we believe that the out-patient clinics at the SCI-center are easily accessible and that persons living with SCI know where to turn for advice. Finally, there are active local peer groups and WhatsApp SCI-groups that together with family provide support for the persons living with SCI.

The living situations were similar to the 2^nd^ follow-up, with the majority being singles however, only two participants lived by themselves. Living independently often requires that you have and can drive an adapted car, which is costly and not an option for most persons with SCI in Botswana. Living in single households in Botswana is not as common as in Sweden, for example, where over 50% are single households [24], however, even though exact numbers are hard to find, the general living situation in Botswana is a mean of 3.8 persons per household [25].

Functional outcomes according to FIM-assessments and QoL showed high ratings, i.e. the participants who survived and were able to attend follow-ups maintained relatively good functional skills and psychological health. Dixon et al. [26] found that QoL ratings were higher 10 years after injury as compared with 18 months, however due to our small population, the non-significant statistical analysis is uncertain. Other studies have shown high rates of depression [3, 23] which is likely to affect many factors of life. Better psychological health can have contributed to the decreased rates of morbidity, since it is well known that depression can affect PU in a negative direction [1, 23].

When it comes to the work situation, persons with disabilities are often seen as another mouth to feed without being able to contribute to the family’s finances [5] and return-to-work is often hindered by inaccessibility in the society and lack of transport [9], increasing the risk of poverty and pre-mature death [1, 3]. In this present study almost half of the participants were working or were students which is in line with a recent review [27]. One participant had recently stopped working after he was laid off by the employer. In Botswana there are no laws protecting persons with disabilities in work related issues, and people can be dismissed on medical grounds.

Pre-mature mortality in general, is reported to be high in the southern part of Africa [2, 22]. In our study, the mortality rate was 26%, with the addition that four persons were lost to follow-up. This was somewhat higher than expected after having 100% survival from discharge to two years post-SCI [17] and might also indicate increased awareness among persons with SCI in seeking attention from the unit when needed and that it takes time to learn how to adjust to the preventive strategies needed after SCI. Considering the estimated high mortality rate before the establishment of the SCI-center, approximately 85% among wheelchair users within a year from discharge according to the local doctor who has work with this patient group for many years, survival has greatly improved.

Septicaemia due to PU is a common cause of mortality in low-resource settings and was also the main cause of death in this study. For at least seven of the 10 participants who had deceased, causes of mortality were likely SCI-related, due to PU, autonomic dysreflexia, or collapse. Identifying autonomic dysreflexia as the potential cause were done by the medical staff at the unit from the description from the relatives. Sweating, headache, and high blood pressure had been present probably due to bladder problems. These outcomes strengthen the need for increased knowledge also at local clinics when persons with SCI live far away from SCI-clinics. The medical staff identified that cardiovascular complications can be possible causes for the lethal collapses that occurred, due to the increased risk among the SCI-population [14].

All but two participants deceased in their homes and no autopsies were conducted resulting in no causes of mortality being recorded in the hospital. Information of cause and time of death was provided by the persons’ families who contacted the SCI-center staff and should therefore be viewed as more anecdotal. Øderud [3] describe a more robust method to gather information regarding causes of death in areas where civil registration and death certification systems are weak. This more robust method is done as a verbal autopsy through structured interviews with the relatives or other caregivers and is often the only way to establish causes of death in these kinds of settings [28]. At the SCI-center in Botswana, these interviews have not been used, but might be implemented in the future to strengthen the reliability of mortality information. However, we still believe that it is appropriate to include the mortality data since they provide potential causes of mortality in a middle-income setting. The unit has previously no structured documentation of mortality data however, the need for this has been clarified and documentation of retrieved information regarding mortality will be initiated for future studies.

### Strength and weaknesses

A major strength of this study is that it has been conducted in the southern part of Africa from where data is largely lacking. Persons with TSCI in Botswana are being largely followed-up and data are documented in patient files. For those who attended follow-up visits, data were mainly complete. However, the weaknesses of the study include lack of data especially from those who did not attend follow-up visits or are lost to follow-up. Often the last recorded data in their patient files were from their discharge notes, i.e. approximately 9–10 years old. Additionally, circumstances around fatalities are vague due to that accurate data were not known or documented. Data for comparison were retrieved from their last follow-up visits, however medical files were lost for four patients and for those no data is available. The missing data and the small sample results in limited and unreliable statistical comparisons.

## Conclusion

This was a follow-up study at year 10 after acute TSCI in Botswana conducted at the national SCI-rehabilitation center. The study supports previous reports regarding the importance of that having specialized SCI units for this patient group and the need of structured regular follow-ups, a responsible person in charge of scheduling patients, and an updated patients register. In this study we found high follow-up rate, low rates of deadly complications, and low numbers of patients being lost to follow-up.

## Data Availability

Deidentified data are available from the corresponding authors upon reasonable request.
